# Identification of a novel intronic enhancer responsible for the transcriptional regulation of *musashi1 *in neural stem/progenitor cells

**DOI:** 10.1186/1756-6606-4-14

**Published:** 2011-04-13

**Authors:** Satoshi Kawase, Takao Imai, Chikako Miyauchi-Hara, Kunio Yaguchi, Yoshinori Nishimoto, Shin-ichi Fukami, Yumi Matsuzaki, Atsushi Miyawaki, Shigeyoshi Itohara, Hideyuki Okano

**Affiliations:** 1 Department of Physiology, Keio University School of Medicine, 35 Shinanomachi, Shinjuku, Tokyo, Japan; 2 Department of Biophysics and Biochemistry, Graduate school of Sciences, Tokyo Medical and Dental University, Tokyo, 113-8510, Japan; 3 Laboratory for Behavioral Genetics, Brain Science Institute, RIKEN, Saitama, 351-0198, Japan; 4 Laboratory for Cell Function Dynamics, Advanced Technoloty Development Group Brain Science Institute, RIKEN, Saitama, 351-0198, Japan; 5 Life Function and Dynamics, ERATO, JST, Saitama, 351-0198, Japan

## Abstract

**Background:**

The specific genetic regulation of neural primordial cell determination is of great interest in stem cell biology. The Musashi1 (Msi1) protein, which belongs to an evolutionarily conserved family of RNA-binding proteins, is a marker for neural stem/progenitor cells (NS/PCs) in the embryonic and post-natal central nervous system (CNS). Msi1 regulates the translation of its downstream targets, including *m-Numb *and *p21 *mRNAs. *In vitro *experiments using knockout mice have shown that Msi1 and its isoform Musashi2 (Msi2) keep NS/PCs in an undifferentiated and proliferative state. Msi1 is expressed not only in NS/PCs, but also in other somatic stem cells and in tumours. Based on previous findings, Msi1 is likely to be a key regulator for maintaining the characteristics of self-renewing stem cells. However, the mechanisms regulating *Msi1 *expression are not yet clear.

**Results:**

To identify the DNA region affecting *Msi1 *transcription, we inserted the fusion gene *ffLuc*, comprised of the fluorescent *Venus *protein and firefly *Luciferase*, at the translation initiation site of the mouse *Msi1 *gene locus contained in a 184-kb bacterial artificial chromosome (BAC). Fluorescence and Luciferase activity, reflecting the *Msi1 *transcriptional activity, were observed in a stable BAC-carrying embryonic stem cell line when it was induced toward neural lineage differentiation by retinoic acid treatment. When neuronal differentiation was induced in embryoid body (EB)-derived neurosphere cells, reporter signals were detected in Msi1-positive NSCs and GFAP-positive astrocytes, but not in MAP2-positive neurons. By introducing deletions into the BAC reporter gene and conducting further reporter experiments using a minimized enhancer region, we identified a region, "D5E2," that is responsible for *Msi1 *transcription in NS/PCs.

**Conclusions:**

A regulatory element for *Msi1 *transcription in NS/PCs is located in the sixth intron of the *Msi1 *gene. The 595-bp D5E2 intronic enhancer can transactivate *Msi1 *gene expression with cell-type specificity markedly similar to the endogenous Msi1 expression patterns.

## Background

Neural stem cells (NSCs) are one of the most important research targets in developmental neurobiology, and are attracting attention in strategies for central nervous system (CNS) regeneration [1-6]. NSCs are somatic stem cells that exist in both the embryonic and adult CNS, and they can be defined conceptually as cells that possess both multipotency and the ability for self-renewal [1,2]. Selective NSC markers include the intermediate filament protein Nestin and the RNA-binding protein Musashi1 (Msi1) [7,8]. In the mammalian embryonic CNS, neural stem/progenitor cells (NS/PCs), which include NSCs and neural precursor cells, are present in the ventricular zone of the developing neural tube. Immunoreactivity against Nestin and Msi1 are consistently detected in the ventricular zone [7-9].

The Musashi family of RNA-binding proteins [10,11] is evolutionarily conserved. Two members, Msi1 and Msi2, have been identified in mammals [8,10,12]. Furthermore, the expression pattern of Msi1 was investigated using a specific monoclonal antibody against Msi1 [9]. Msi1 is downregulated in post-mitotic neurons in the course of neural differentiation [8]. Msi1 is believed to contribute to maintaining the stemness of NS/PCs in the embryonic and post-natal stages through the translational regulation of its target mRNAs, which are involved in regulating cell fates and the cell cycle [10,11,13]. We have identified Musashi-binding RNA sequences in mammals [14] and *Drosophila *[15]. Our previous studies revealed that Msi1 contributes to NS/PC maintenance by binding to the 3'-untranslated region (UTR) of *m-numb *mRNA, an Msi1 target, and repressing its translation [14]. The *m-numb *mRNA encodes a membrane-associated protein that inhibits Notch signalling [16]. Other Msi1 target mRNAs and regulatory pathways have also been reported [17-19]. Interestingly, some groups have reported that high Msi1 expression potentiates Notch signalling or causes cell-cycle progression in certain tumour cells [20-22]. Similarly, Msi1 is known to be associated with various kinds of tumours, including glioblastoma, hepatoma, and intestinal tumours [23-25].

Recently, mammalian Msi1 protein was identified not only in the CNS, but also in other tissues and organs. Intriguingly, Msi1 has been detected in somatic stem cells in adult tissues, including the eye [26], intestine [27], stomach [28], mammary gland [29], hair follicle [30], and germ-line tissue [31]. Thus, Msi1 may contribute to maintaining the stem cell state by controlling the translation of downstream target genes. To better understand Msi1's spatial and temporal distribution in NSCs, we sought to identify the genome region involved in *Msi1 *gene transcription. Furthermore, the identification of this region may lead to putative transcription factors involved in regulating Msi1's expression.

Relationships between stem cells and transcription factors in the CNS have been intensively investigated [32,33]. For example, members of the SOX (Sry like HMG box protein) family are known to be transcription factors that define characteristics of stem cells, including NSCs [34]. SOX2 is expressed in embryonic stem cells (ESCs), and both SOX1 and SOX2 are expressed in NS/PCs after neural induction [35]. SOX proteins are known to be involved in the transcriptional activation of the *Nestin *gene. The *Nestin *NSC-specific transcription enhancer is present in its second intron and contains binding sites for SOX and POU (Pit-Oct-Unc)[36,37]. The POU family was originally defined as transcription factors containing a common region called a POU domain. Oct4, a member of the POU family, plays a role in regulating Sox2 in inner-cell-mass ESCs and during their transition to neural cells, before the recruitment of neural POU factors such as Brn1 and Brn2 [36-38]. Indeed, SOX2 and Brn2 cooperatively transactivate *Nestin *gene transcription through the second intron enhancer [39]. While the mechanism of *Nestin *transcription has been clarified, those of other NSC markers, including the *Msi1 *gene, remain unclear.

There has been tremendous innovation in the last decade in scientific engineering techniques, materials, and knowledge databases, including genome sequence databases. In particular, the science of genome information has progressed not only in regard to nucleotide sequence information, but also in the procedures for analyzing chromatin modification and transcription-factor binding. The development and dissemination of bacterial artificial chromosomes (BAC) enables the use of genomic DNA, including large areas that can cover the whole locus of a gene [40-43]. Engineering techniques such as DNA recombination methods have also been improved. Fluorescent protein reporter genes have been developed and improved [44-47]. Taking advantage of these tools, we constructed a recombinant BAC reporter to analyze the molecular mechanism of *Msi1 *transcription regulation. We used a homologous recombination technique to insert the newly generated reporter gene *ffLuc *near the *Msi1 *transcription start site (TSS). Using this reporter-containing BAC, which we designated *Msi1-ffLuc*, we were able to analyze where *Msi1 *transcription is activated. Through these investigations, we have identified a new Msi1 transcription enhancer element in NS/PCs. This region is located in the 595-bp region within the sixth intron of the *Msi1 *gene, and contains SOX- and AP-2-binding sites.

## Results

### Generation of an *Msi1-*transcription reporter BAC bearing a 184-kb *Msi1 *genomic region

**Figure 1:**
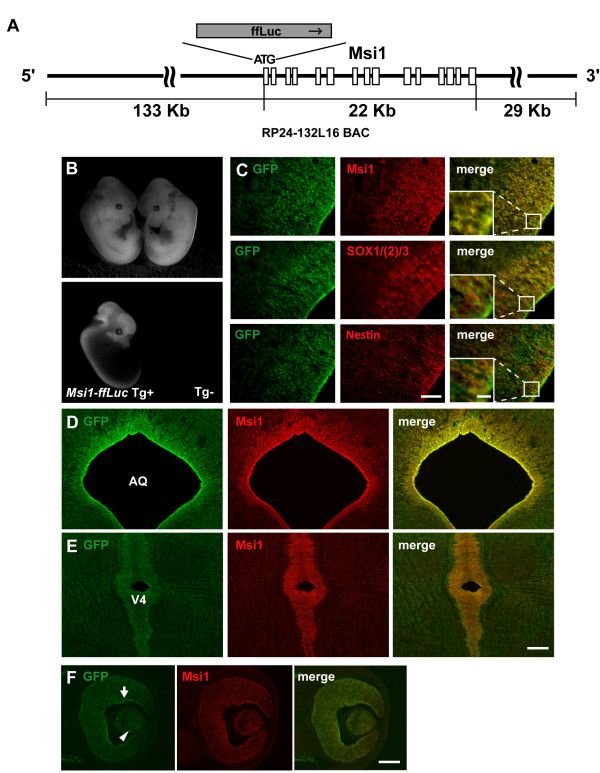
**The 184-kb *Msi1-*reporter expression corresponds to endogenous Msi1 expressed in NS/PCs *in vivo***. (A) The original BAC (RP24-132L16) and the position of *Msi1 *are shown. There was a 22-kb region of *Msi1 *exons and introns between the 133-kb 5' upstream and 29-kb 3' downstream regions of *Msi1*. The translation initiation codon (ATG) was replaced with *ffLuc *to produce *Msi1-ffLuc*. (B) GFP in the E12.5 transgenic mouse containing *Msi1-ffLuc*, was expressed in the central nervous system and eye (upper panel: bright-field image, lower panel: fluorescent image). (C) Immunohistochemical analysis in the E14.5 Tg mouse cortex. Msi1, Group B1 SOX [SOX1/(2)/3], and Nestin expression in undifferentiated cells in the ventricular zone co-localized with GFP expression (Scale bar: 50 μm). The images shown in the small boxes in the right column panels are magnified and shown in the left corner boxes (Scale bar in magnified image: 10 μm). GFP was also present in the ventricular zone of the midbrain (D), pontine area (E), and eye (F). An arrow shows expression of GFP in retinal ganglion cell layer and an arrowhead shows expression of GFP in lens. AQ: aqueduct of midbrain, V4: fourth ventricle. Scale bar: 100 μm.

To identify transcription or signalling pathway-associated factors involved in regulating *Msi1 *gene transcription, we generated a BAC reporter gene to detect *Msi1 *transcriptional activity in NS/PCs. We previously reported generating an *Msi1 *transcription reporter gene that contained an approximately 3-kb 5' upstream region of the mouse *Msi1 *TSS, which, through unknown mechanisms, could act in human cells but not in mouse cells [48]. To elucidate *Msi1 *transcriptional regulatory mechanisms *in vivo *more precisely, we prepared the RP24-132L16 BAC as a genomic element containing many of the *cis*-elements that direct *Msi1 *transcription. As shown in Figure 1A, the RP24-132L16 BAC contains 133-kb of the 5' region upstream of the *Msi1 *TSS, the *Msi1 *mRNA coding region (exons-introns coding region), and 29-kb of the 3' region downstream of the 3'-end of the *Msi1 *gene. We then used homologous recombination techniques to insert the *ffLu*c reporter gene into the *Msi1 *translational initiation site of the RP24-132L16 BAC (Figure 1A). The *ffLuc *reporter gene encodes a fusion protein of the fluorescent protein Venus and firefly Luciferase [45,46]. This reporter gene both visualized *Msi1 *transcriptional activity *in vivo*, and allowed us to use Luciferase bioluminescence to quantify the level of transcriptional activity.

### Generation of *Msi1-*transcription reporter BAC tg mice and recombinant ES cell lines

To determine how well the *Msi1 *BAC reporter gene expression reflected endogenous Msi1 protein expression *in vivo *both spatially and temporally, we generated *Msi1 *BAC reporter transgenic (tg) mice. GFP fluorescence was observed in the CNS of embryonic day (E) 12.5 *Msi1 *reporter tg mice, while it was not detected in wildtype littermates (Figure 1B). For the rest of this article, we will describe Venus fluorescence as GFP fluorescence, and Venus expression immunodetected with anti-GFP antibody as GFP expression. We next used immunohistochemistry to analyze the distributions of GFP, Msi1, Group B1 SOX, and the intermediate filament protein Nestin in the cerebral cortex of E14.5 mice. We observed that GFP was expressed in the ventricular zone, where proliferative NS/PCs are present (Figure 1C, D, E). GFP was also expressed in the retinal ganglion cell layer, neuroblastic layer and lens in the developing eye (Figure 1 F). GFP expression co-localized with endogenous Msi1, Group B1 SOX, and Nestin (Figure 1C). GFP expression was also observed in the subventricular and subgranular zones of the adult mouse brain, where it co-localized with endogenous Msi1 (Additional file 1, Figure S1). Based on these observations, we concluded that the *Msi1 *BAC reporter can reflect endogenous Msi1 expression in the CNS *in viv*o.

**Figure 2:**
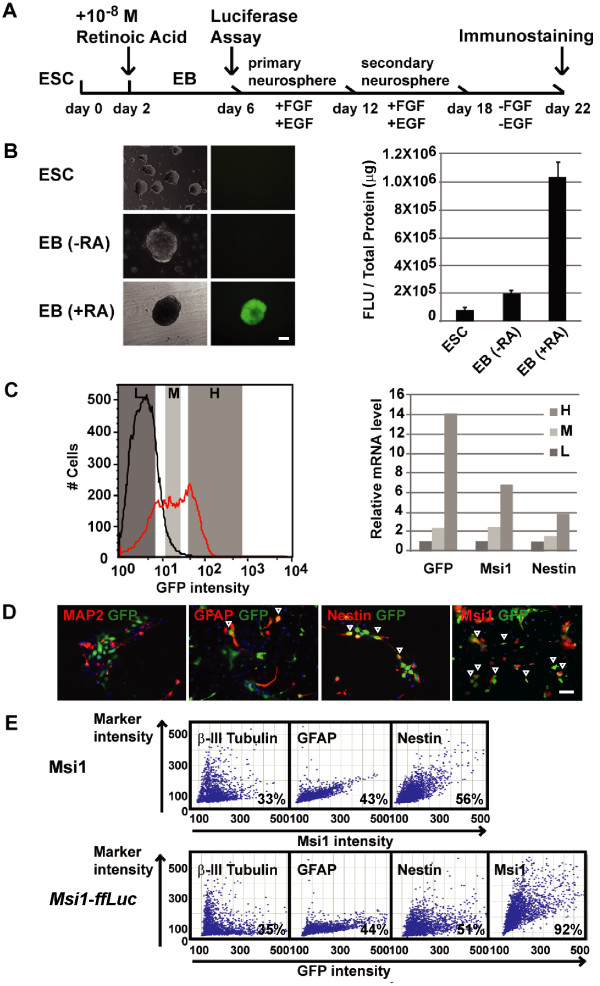
***Msi1-ffLuc *expression corresponds with Msi1-positive NS/PCs and astrocytes in *Msi1-ffLuc *ESC-derived neural cells**. (A) The experimental protocol for embryonic stem cell (ESC) differentiation with a retinoic-acid (RA) conditioned medium. ESCs formed embryoid bodies (EB) after 6 days in floating culture. (B) The expression of *Msi1-ffLuc *on day 0 of ESCs and day 6 of EBs is shown in the left panels. GFP was not expressed in ESCs and RA-untreated EBs(-RA) but was expressed in RA-treated EB(+RA). Nine *Msi1-ffLuc *ES cell lines were analysed for GFP fluorescence in each condition indicated (right panel). Scale bar: 100 μm. FLU: firefly luciferase light unit. (C) Dissociated day-6 EBs (+RA) derived from *Msi1-ffLuc *ES cell line (Red line in left panel) were assessed by flow cytometry and divided into H, M, and L fractions according to their GFP intensity. Each fraction was then analysed by qRT-PCR. The GFP, Msi1, and Nestin mRNA transcripts were enriched in the H fraction (L = 1 in each fraction). Black line in left panel shows day-6 EBs (+RA) derived from wild type ES cell line. (D), (E) secondary neurospheres were dissociated, cultured in differentiation medium for 4 days, and immunostained. Anti-GFP reactivity for GFP(+) cells correlated with Nestin(+) and endogenous Msi1(+) cells, but not with MAP2(+) mature neurons with long processes (D, Scale bar: 50 um). Vertical arrowheads show Marker(+)/GFP(+) cells. The immunofluorescent intensity of each marker was represented by scatter blots (E). The vertical axis shows the intensity of Msi1 and of cell-type-specific markers for neurons (β-III Tubulin), astrocytes (GFAP) and NS/PCs (Nestin). The horizontal axis shows the endogenous Msi1 (upper panel) and GFP (lower panel) intensities. The proportion (%) of marker(+) cells among GFP(+) cells is indicated in the bottom of each box. Note that the GFP/Marker double-positive cells were highly observed for Msi1, GFAP, and Nestin, but the β-III Tubulin-positive neurons had weak GFP expression.

We next examined how well the BAC reporter signal corresponds to endogenous Msi1 expression during the neural differentiation of ESCs. For this purpose, we generated ESC lines with stably-integrated *Msi1 *BAC DNA. We introduced *Msi1 *BAC DNA into parent (line EB3 tg14) ESCs. Forty-seven stable lines were established and characterized by their neural differentiation, using the scheme shown in Figure 2A. ESCs can be induced into neural lineages, including NSCs, by treatment with retinoic acid (RA) at a low concentration (10^-8^M). In embryoid body (EB)-forming cells, NS/PCs expressing Msi1 and/or Nestin can be efficiently induced by treatment with 10^-8^M RA [49]. We cultured recombinant ESCs in floating conditions to form EBs, added 10^-8^M RA to the culture medium after 2 days to induce neural differentiation, and cultured the cells for 4 more days (Figure 2A). Recombinant ES clones bearing *Msi1 *BAC DNA frequently showed GFP fluorescence on EB day 6 with RA (+RA), while GFP was not expressed in ESCs and RA-untreated EBs(-RA) (Figure 2B left panel). Firefly Luciferase activity, reflecting the *Msi1 *transcription activity, was measured in EBs treated with RA and without RA. The average luminescent activity per total protein increased about 10-fold in the treated EBs (+RA) over that found in undifferentiated recombinant ESCs (Figure 2B right panel). Luciferase activity in treated EBs (+RA) was about 5-fold greater than in untreated EBs (-RA) (Figure 2B right panel).

To determine whether the GFP fluorescence correlated with *Msi1 *mRNA levels, we determined the *Msi1 *mRNA levels in three cell populations (H, M, and L). To form these populations, we dissociated EB cell aggregates derived from representative recombinant ES lines, and separated them by cell sorter based on their fluorescence intensity (H > M > L) (Figure 2C, left panel). As we expected, the *GFP *mRNA level, as measured by quantitative RT-PCR, was in proportion to the GFP fluorescence, and the *Msi1 *and *Nestin *mRNA levels were also in proportion to the GFP fluorescence (Figure 2C, right panel). Taken together, these findings show that the *Msi1 *mRNA level increased during induced differentiation toward NSCs, as reflected in the Luciferase activity, and that the *Msi1 *mRNA level correlated with the intensity of GFP fluorescence. In addition, we used flow cytometric analysis to examine whether the GFP and Msi1 levels were correlated. Of all the EB (+RA) cells, 89% were positive for Msi1, and 80.5% were positive for both GFP and Msi1 (Additional file 2, Figure S2), indicating that the GFP expression correlated well with the Msi1 expression.

Next, we characterized the cell-type specificity of the GFP-expressing cells by immunochemical analysis. After 4 days in RA-treated culture, the EBs were dissociated and placed in floating culture conditions in the presence of FGF-2 and EGF; primary neurospheres formed after 6 days of culture (Figure 2A). The primary neurospheres were dissociated, and secondary neurospheres were subsequently generated in the same culture conditions for an additional 6 days. The secondary neurospheres were then dissociated and induced into neuronal and glial differentiation in an attached condition, without FGF-2 or EGF. After 4 days in differentiation culture, few cells expressing the post-mitotic neuronal marker protein MAP2 demonstrated immunoreactivity against GFP. However, Msi1 and Nestin were co-localized with GFP (Figure 2D).

We quantified the overlap of Msi1 with GFP and with differentiation markers by using high-throughput analyzing microscopy with the In Cell Analyzer 2000 (GE Healthcare Biosciences). We observed that cells expressing GFAP, Nestin, and βIII-Tubulin corresponded to 43%, 56%, and 33% of the endogenous Msi1-expressing cells and that Msi1 expression level was weak in βIII-Tubulin-positive cells (Figure 2E upper left panel). Some of cells were positive for both Msi1 and GFAP or βIII-Tubulin. These observations are consistent with previous findings that Msi1 is expressed in GFAP-positive astrocytes, and that low levels of Msi1 are observed in young, immature neurons [9,12]. Significantly, cells expressing Msi1, GFAP, Nestin, and βIII-Tubulin corresponded to 92%, 44%, 51%, and 35% of the GFP-expressing cells, similar to endogenous Msi1 positive cells (Figure 2E lower panel). It is especially noteworthy that Msi1(+) cells correlated well with GFP(+) cells (Figure 2E lower panel). Thus, Msi1-expressing cells correlated well with GFP-expressing cells in a neural differentiation system using ESCs bearing the *Msi1 *BAC reporter.

### Deletion study of the *Msi1 *BAC reporter to identify *cis*-elements involved in *Msi1 *gene transcription

**Figure 3:**
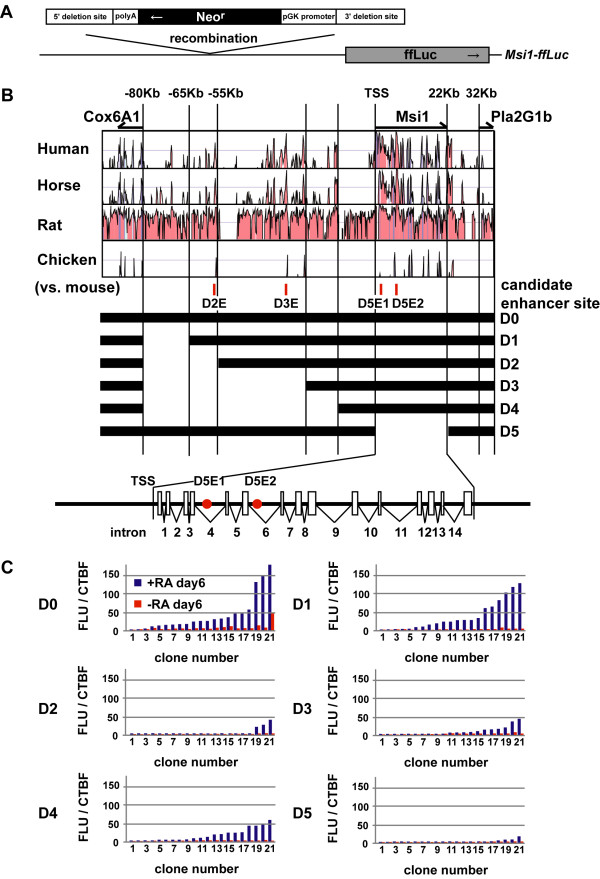
***Msi1 *BAC deletion studies suggest that both the 55-65 kb 5' upstream region and the exon-intron coding region have *Msi1 *transcriptional activity**. (A) Schematic representation of our deletion method. Targeted *Msi1 *enhancer/promoter regions were replaced with *pgk pro.-Neo*^*r*^*-bGH pA *to select for successfully recombined clones. (B) The conserved genomic region between *cox6a1 *and *pla2g1b *in *Msi1-ffLuc *is shown. VISTA plots comparing the alignment of mouse versus human, horse, rat, and chicken. Full-length *Msi1-ffLuc *(D0) and deletion constructs (D1-D5) are also indicated. Blanks represent deleted regions replaced with *pgk pro.-Neo*^*r*^*-bGH pA*. The positions of candidate enhancer sites used in later experiments are also shown (see details in Results). The exon-intron coding region of *Msi1 *gene is magnified. Red circles indicate candidate enhancer sites, white boxes indicate exons. (C) Gene expression in EBs derived from the deletion-reporter ES cell lines. All 21 cell lines were analysed for Luciferase expression in +RA neural induction cultures for 6 days (blue bars). Clones are presented in rank order for neural cultures. The red bars show the activity in the same clone under non-neural inducing conditions (-RA, not in rank order). Luciferase activity was normalized to the cell-viability fluorescence intensity using CellTiter-Blue (CTBF). The *Msi1 *expression in D0 and D1 was greatly increased under the neural-inducing conditions. Note that D5 showed decreased *Msi1 *expression; D2-D4, which included a 55-65-kb deletion, showed an even greater decrease.

To elucidate the regulatory mechanisms of *Msi1 *gene transcription, we performed a deletion study to determine whether there are *cis*-elements in the 184-kb region of genomic DNA containing the *Msi1 *loci that are sufficient for inducing *Msi1 *transcription. Accordingly, a module containing the neomycin-resistance gene was inserted into the *Msi1 *5'-upstream region and the exon-intron coding region (D1-D5). The BAC constructs were generated by homologous recombination, as shown in Figure 3A and 3B. The length and location of the deleted regions were designed by considering evolutionarily conserved regions among species--mouse, human, horse, rat, and chicken. This homology alignment was performed with the VISTA homology search program [50]. It was also taken into consideration that the adjacent genes Cox6A1 and Pla2G1b are located 80-kb upstream and 54-kb downstream of the *Msi1 *TSS, respectively (Figure 3B).

The deleted BAC constructs were introduced into ESCs, and stable transformants were established for each BAC reporter construct. In a similar manner to the RA treatment and Luciferase reporter assay conducted with EBs (shown in Figure 2A), the *Msi1 *transcriptional activities were quantified using the deletion-containing reporters D1-D5 and the full-length BAC reporter D0. The Luciferase activity was measured by normalization with CellTiter-Blue [Promega], which was used for a live cell count. The gene expression level of 21 clones for each deletion or the full-length transformant showed that the Luciferase activity was lower in EBs bearing the D2-D4 deletion reporter genes than in those bearing the D1 or the full-length D0 reporter genes (Figure 3C). These results suggested that the 10-kb region from 55 kb to 65 kb upstream of the TSS might contain *Msi1 *transcriptional enhancers, and we named this region the 'upstream 10-kb enhancer.' Furthermore, when the region after the transcription site (exons and introns coding region) was deleted, the *Msi1 *transcriptional activity diminished even in the presence of the 'upstream 10-kb enhancer' (Figure 3C). These results indicated two regions responsible for *Msi1 *transcription. However, the exon-intron coding region is likely to be primarily necessary for *Msi1 *transcription.

To find the region responsible for *Msi1 *transcription, we performed a genome informatics database search. The UCSC Genome Browser on Mouse Feb. 2006 (NCBI36/mm8) Assembly showed that there were approximately three notable H3K4me1 ChIP-sequence tags in the 184-kb BAC-contained region, using neural progenitor cells as material (Additional file 3, Figure S3). The regions corresponding to the three tags are labelled as D2E, D3E and D5E2 in Figure 3B and Additional file 3, Figure S3. Monomethylation, but not trimethylation, of lysine 4 of histone H3 is known as an active enhancer [51]. These three regions were also CpG islands, which indicates that they may be developmentally regulated regions. The first region, designated D2E, was in the upstream 10-kb enhancer region (Figure 3B) and was highly homologous among the five indicated species. The second region was located 31-kb upstream of the TSS, and we designated this as D3E (Figure 3B). The third region was located 7.3-kb downstream from the TSS, which is included in the sixth intron, and we designated this as D5E2 (Figure 3B). Interestingly, D3E and D5E2 were also identified as p300-binding regions in the embryonic forebrain [52,53]. The transcriptional coactivator protein p300 is expressed almost ubiquitously in mouse embryogenesis, and can bind to a wide spectrum of active tissue-specific enhancers. D3E is also a highly homologous region among the mouse, human, horse, rat, and chicken genes. D5E2 exhibits high homology among four of the species, but not with chicken. Of these three regions that could be responsible for *Msi1*'s transcriptional enhancement, the results of our *Msi1 *BAC deletion study (Figure 3) and previous bioinformation studies led us to analyze the enhancer function of two of them, D2E and D5E2, in more detail in NS/PCs.

### Minimized reporter assays in NS/PCs revealed that D5E2 was competent to control *Msi1 *transcription specificity

**Figure 4:**
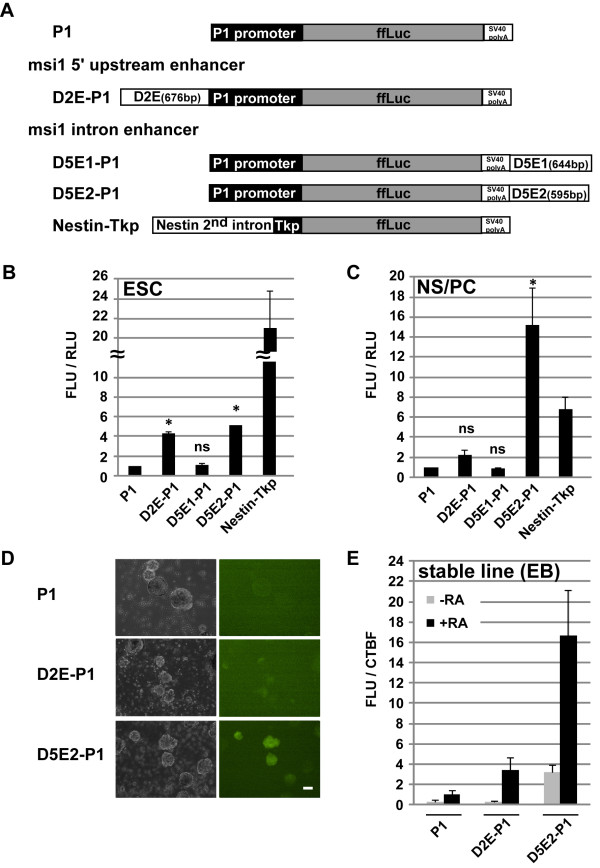
**D5E2 is a transcriptional enhancer that functions in NS/PCs**. Candidate enhancer alignments were selected using information from the UCSC genome data base and by looking at p300-binding sites [52,53] in the expected enhancer regions of introns and 55-65 Kb upstream. Each enhancer reporter gene used in the experiments is shown in (A). Nestin second intron-Tkp was used as a positive control. (B), (C) Candidate enhancer activities in ESCs and NS/PCs. Dissociated E14.5 cortex cells were cultured on a poly-D-Ornithine/fibronectin coated dish in an FGF-2/EGF mixed selective NS/PC conditioned medium. Enhancer reporter constructs were transfected into EB3 tg14 and NS/PCs, and the Luciferase activity was detected after 48 hours. The D2E and D5E2 sites enhanced the transcriptional activity in ESCs, and the D5E2 site enhanced it in NS/PCs [4.3-fold, 5.1-fold in ESCs, 2.2-fold, 15.2-fold in NS/PCs, respectively, compared with P1 (P1 = 1)], but D5E1 could not enhance the activity in either cell type. The data represent the mean ±SEM of three independent experiments. The data were subjected to non-repeated-measures ANOVA tests, and p values were calculated by Bonferroni multiple comparison tests. *p < 0.05: P1 to D2E-P1, D5E1-P1, or D5E2-P1, ns: not significant. FLU/RLU: firefly luciferase light unit/renilla luciferase light unit. (D), (E) The D2E and D5E2 enhancer activities were confirmed in EBs: 47 stable ES cell lines for P1, D2E-P1, and D5E2-P1were established and cultured in EB-formation conditions with or without RA. (D) Day 6 of neural-induced EB (+RA). Left panel shows bright field and right panel shows fluorescent images. Scale bar: 100 μm. (E) All cell lines were analysed for each condition on day 6. Average intensity is shown in comparison to RA-treated P1 (P1: +RA = 1, -RA = 0.33, D2E-P1: +RA = 3.47, -RA = 0.26, D5E2-P1: +RA = 16.7, -RA = 3.18.). Note that D5E2-P1 showed potent enhancer activity in the neural induced EBs.

To investigate whether D2E and D5E2 function as localized enhancer regions to regulate *Msi1 *transcription, we generated constructs containing a minimal enhancer (D2E, D5E2, or none), the P1 promoter 1-kb upstream of the *Msi1 *TSS, and *ffLuc *(Figure 4A). With the original locations on the genome in mind, D2E was placed at the 5'-end of the P1 promoter, and D5E2 was placed at the 3'-end of the *ffLuc-SV40 poly(A) *additional signal element (Figure 4A). D5E1, which was included in the fourth intron and was well-conserved in the mouse, human, horse, and rat species (but not chicken), was not included in the p300 ChIP-sequence tags, and was used as a negative control [53] (Figure 3B). The Nestin-TKp construct contained a rat *Nestin *second intron enhancer combined with a thymidine kinase minimal promoter element, and *ffLuc*. This *Nestin *enhancer is known to induce transcriptional activity in NS/PCs [35,54]. These constructs were transiently introduced with an internal control Renilla luciferase-expressing plasmid into ESCs or NS/PCs derived from the mouse brain cortex at E14.5 and cultured in the presence of FGF-2 and EGF. The transfected cells were lysed after 2 days in culture, and their Luciferase activity was measured. The Luciferase activity was low in ESCs or NS/PCs with the D5E1-P1 construct or with only the P1-promoter construct. We also observed weak Luciferase activity in ESCs with D2E-P1 or D5E2-P1; reporter signals in these ESCs showed 4-5-fold increases compared to the signals in cells with only the P1 construct. Interestingly, the Luciferase activity in NS/PCs with a transfected D5E2-P1 construct increased markedly, 15.2-fold, compared with the signals of cells with the P1 construct. When D2E-P1 was introduced into NS/PCs, a 2.2-fold increase in the reporter signal was observed compared to P1 alone, however, there were no stastical difference between them. These findings together indicated that D5E2 can work efficiently as an *Msi1 *transcription enhancer in terms of the strength and specificity of transactivation in NS/PCs.

We next generated recombinant, stable ES lines containing each of the constructs P1, D2E-P1 and D5E2-P1 to determine whether the D5E2 enhancer has cell-type specificity and transactivation intensity during neuronal and glial differentiation. RA was added to the medium at 10^-8^M concentration 2 days after starting floating EB culture. After an additional 6 days, GFP fluorescent and firefly Luciferase activity were frequently observed in EBs (+RA) derived from ES clones containing the D5E2-P1 construct (Figure 4E). There was no detectable GFP fluorescence or firefly Luciferase activity in the EBs (+RA) derived from ES clones containing the P1 construct (Figure 4E). The GFP expression and Luciferase activity were less intense in the D2E-P1-bearing EBs (+RA) than in the D5E2-P1-bearing EBs; P1 = 1, D2E-P1 (+RA) = 3.4, D5E2-P1 (+RA) = 16.7 (Figure 4D, E). These results indicated that D2E and D5E2 are capable of transactivation in NS/PCs derived from EB (+RA) cells. The transactivation intensity of the D5E2 enhancer was higher than that of the D2E enhancer in NS/PCs, similar to our findings in cultured NS/PCs derived from the mouse embryonic cortex.

### D5E2 is an authentic enhancer that reflects Msi1 transcription activity and directs cell-type-specific transactivation during neurogenesis and gliogenesis

**Figure 5:**
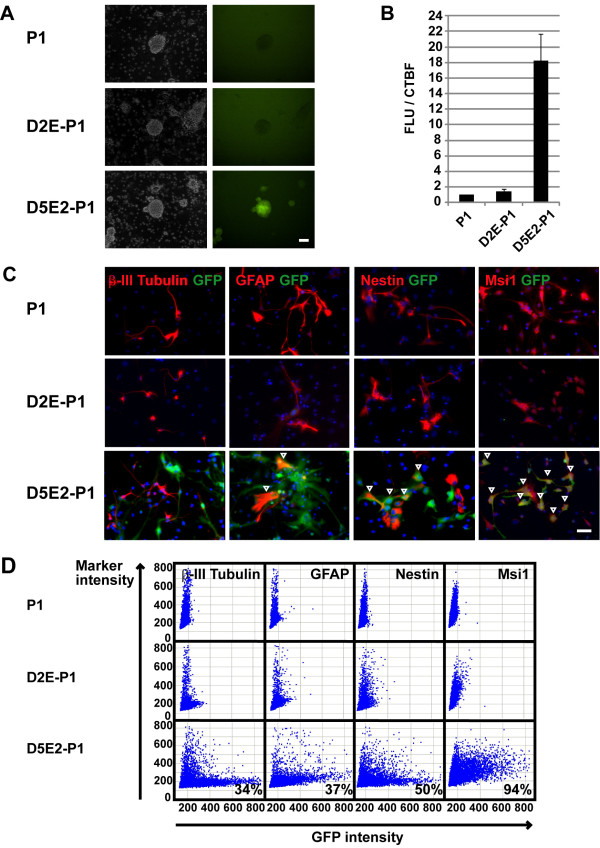
**D5E2-P1 expression corresponds with Msi1-positive NS/PCs and astrocytes**. (A), (B) D5E2-P1 was active in primary neurospheres. (A) Primary neurospheres derived from ESC lines, on day 6. Scale bar: 100 μm. (B) Three cell lines with intense reporter expression were selected from 47 ES cell lines; EB(+RA) were selected and primary neurospheres were formed in each clone. The reporter activity was analysed on day 6. (C) Cells from secondary neurospheres were differentiated and stained with each antibody shown. D5E2-P1 showed intense GFP expression. GFP was not induced by P1, and was only slightly induced by D2E-P1. Vertical arrowheads show Marker(+)/GFP(+) cells. D5E2-P1 induced GFP expression in Msi1(+) and Nestin(+)/GFAP(+) cells. Scale bar: 50 um. (D) Immunofluorescence intensity was visualized by scatter plots. The vertical axis shows the intensity of Msi1 and each cell-type-specific marker. The horizontal axis shows GFP intensity. The ratio of marker(+) GFP(+)/GFP(+) (%) cells is indicated at the bottom of the boxes.

We next examined whether the activities of the minimized *Msi1 *transcriptional enhancers corresponded with endogenous Msi1 expression, which was high in NS/PCs and astrocytes and low in the neuronal linage. For this purpose, EB (+RA)s were taken from the three cell lines showing the most intense Venus expression for each integrated construct (P1-, D2E-P1- and D5E2-P1-integrated EBs) and placed in floating culture for 6 days to form primary neurospheres (Figure 4). GFP-fluorescence was detected in primary neurospheres containing the D5E2 construct (Figure 5A). Primary spheres containing P1, D2E-P1, or D5E2-P1 were also subjected to Luciferase assays. The Luciferase activity was 18.2-fold stronger in the primary neurospheres with D5E2-P1 than in those with P1 alone (Figure 5B), and 1.4-fold greater in the primary neurospheres with D2E-P1 than in those with P1 alone (Figure 5B).

Next, the secondary neurospheres containing minimal reporter genes were dissociated, and the cells were cultured for neuronal and glial differentiation in the same conditions as in the experiment shown in Figure 2A. After 4 days of differentiation culture, the cells were fixed and immunostained with anti-GFP, anti-Msi1, anti-βIII-Tubulin, anti-GFAP, and anti-Nestin antibodies. GFP expression was found in the cells with D5E2-P1, but not in those with P1 alone (Figure 5C). We then quantified the rates of cells positive for Msi1 or other differentiation markers in the GFP(+) population using an In Cell Analyzer 2000. The proportions of GFP(+) cells expressing Msi1, GFAP, Nestin, and βIII-Tubulin were 94%, 37%, 50%, and 34%, respectively (Figure 5D). Notably, the specificity of the reporter signal displayed by the full-length BAC reporter (*Msi1-ffLuc*) was very similar to that of the D5E2-containing reporter. Taking all these results together, we concluded that we had identified one of the regions responsible for *Msi1*'s transcriptional activation: our findings indicate that *Msi1 *transcriptional activity is conserved in 595 bp of the D5E2 region in the sixth intron.

## Discussion

In this study, we generated the reporter gene *Msi1-ffLuc*, which has *Msi1 *gene locus contained in a 184 kb BAC, and confirmed that this reporter accurately reflected endogenous Msi1 expression in the CNS of transgenic mice and in ES-derived neural cells. This reporter makes it possible to detect Msi1 expression by fluorescence and luminescence. The proportion of cells expressing the markers βIII-Tubulin, GFAP, and Nestin in the GFP(+) population correlated well with those among Msi1(+) cells in differentiated EB-derived neural cells, and the fluorescence distribution approximated that for each marker (Figure 2D, E). While almost all the GFP(+) cells were positive for Msi1, strong GFP expression was not observed in MAP2(+) mature neurons. Notably, 35% of the GFP(+) cells (33% of the endogenous Msi1(+) cells) were βIII-Tubulin(+) young neurons. However, the fluorescent intensity of GFP or Msi1 in the young neurons was much weaker than that in GFAP-, Nestin-, and Msi1-positive cells. These findings corresponded with the endogenous Msi1 expression reported previously; Msi1 expression is strongly detected in 30-40% of the cells with the *Tα1 *promoter driving reporter expression, and weakly expressed in βIII-Tubulin(+) or MAP2(+) post-mitotic neurons [9]. We previously found that Msi1 is not detected in mature neurons with long, thick processes; GFP fluorescence was not observed in these cells in the present study either, suggesting that neuronal maturation correlates with a downregulated reporter signal; this is consistent with another report using a *Nestin second intron*-EGFP mouse [54]. GFP-expressing cells were widely present in the ventricular zone through the caudal-rostral axis in the embryonic stage, and were restricted to the subventricular zone and subgranular zone, and to GFAP-positive astrocytes in adult mice. This is similar to Msi1 expression in the adult mouse, and the spatio-temporal regulation is the same as in our findings (Figure 1, Additional file 1, Figure S1) [8,55]. These results indicate that the *Msi1-ffLuc *reporter expression accurately mimics endogenous Msi1 expression, and that this 184-kb genomic region is sufficient to activate *Msi1 *transcription.

We found two regions that increased the *Msi1 *transcriptional activity, in what we designated the "upstream 10-kb enhancer" (55-65 kb region upstream of the TSS) and in the *Msi1 *exon-intron coding region. Luciferase assays of deletion constructs in the absence of each region showed significant Luciferase reduction in EBs(+RA); in particular, almost all the Luciferase activity was diminished in D5. This result indicated that the exon-intron coding region includes requisite enhancer sites. Intense activity through D5E2 enhancer, which is included in sixth intron, supported this conclusion. The upstream 10-kb enhancer site was also an active enhancer region in NS/PCs, although the expression level was low. We prepared a 10-kb enhancer-P1 reporter gene and detected the Luciferase activity in NS/PCs derived from the E14.5 mouse cerebral cortex. A 3.7-fold enhancement was observed over using a reporter gene with P1 alone (data not shown). This increase is subtle compared to the 15.2-fold enhancement seen with D5E2, and the just-over-2.2-fold enhancement with D2E. D2E is one of the most highly evolutionarily conserved sites in the upstream 10-kb enhancer region; no other highly conserved site could be found in this area. D2E was most active in ESCs (4.3-fold), with less activity in EBs(+RA) (3.4-fold) or primary neurospheres (1.39-fold), with the direction of neural lineage. Consistent with our results, p300 does not bind to D2E in either the forebrain or midbrain, while it binds strongly to D5E2 in both regions [52,53]. EBs consist of heterogeneous cell populations even when induced under 10^-8 ^M RA; that is, endodermal cells, mesodermal cells, and ectodermal cells are all present [49]. Msi1-positive cells are found in the intestine, stomach, mammary gland, and germ line. Therefore, the upstream 10-kb enhancer may act in other germ layers outside the neuroectoderm.

D5E2 showed activity in ESCs (5.1-fold) and had higher activity in EBs(+RA) (16.7-fold) and in primary neurospheres (18.2-fold), with the direction of NS/PCs, while D2E showed the opposite pattern. We can recapitulate the temporal regulation of CNS development *in vitro *by using a neurosphere-based culture system of ESC-derived NS/PCs [56]. D5E2 was continuously active in secondary and tertiary neurospheres (TNS) (Additional file 4, Figure S4), indicating that transcription through D5E2 was constantly active in NS/PCs in neural development. To determine whether D5E2 functions bi-directionally as an enhancer, we made a reporter gene with a reverse-oriented D5E2 strand (Additional file 5, Figure S5). The expression level with the reversed strand decreased by half (0.56-fold vs. D5E2-P1 in NS/PCs), but the reversed-oriented D5E2 was significantly still active. This finding supports the identification of D5E2 as an enhancer.

Using D5E2-P1 reporter gene, strong GFP(+) cell populations were observed in GFAP(+) astrocytes or Nestin(+) NS/PCs, and weak GFP(+) populations were observed in βIII-Tubulin(+) neurons. Almost all GFP(+) cells were Msi1(+). When comparing D5E2-P1 with *Msi1-ffLuc*, the GFP(-) cell populations in the Nestin(+) or GFAP(+) cells were slightly increased. This result indicates that the D5E2-induced expression did not represent all of the *Msi1-ffLuc *expression. Previous observations showed that 10^-8^M RA induces wide ranges of regionalized cell populations in the brain--in the forebrain, midbrain, and hindbrain, but not in the spinal cord [49]. In the present experiments, D5E2 was significantly active in EBs (+RA), but regional specificity was not clear, even though p300 binds to D5E2 both in the forebrain and midbrain (52, 53). Transgenic mouse assays with D5E2 will be necessary to resolve this question.

We cannot exclude the possibility that other enhancers activate *Msi1 *transcription after EB day 6, since we screened active enhancer regions on EB day 6 by deletion study of *Msi1*-BAC reporter gene. In addition, in EB lysates that were analysed by Luciferase assay, small cell populations were uncovered in which GFP was expressed by other enhancers.

We searched for transcriptional factor binding sites in D5E2 by *in silico *analysis, and found three well-conserved SOX-binding sites (Additional file 6, Figure S6). SOX family proteins are important for NSC maintenance [57,58], and transcribe the *Nestin *gene cooperatively with POU family genes [39,59]. However, there is no highly matched sequence with a POU-binding consensus in D5E2. A SOX-binding site alone is not sufficient to exert regulatory function; it requires a second site nearby to bind a partner protein that will cooperate with the SOX proteins [60]. Thus, SOX might act with other binding partners if it functions at this enhancer site. Another candidate transcription factor is AP-2. We found four AP-2-binding sites in D5E2. AP-2 is a retinoic acid-inducible transcription factor that is expressed in the nervous system and in neural crest cell lineages during murine development [61,62]. AP-2 proteins can be viewed as gatekeepers, controlling the balance between proliferation and differentiation during embyogenesis [63,64]. From these reports, the expression pattern and functional properties of Msi1 could be relevant to those of AP-2.

Transcriptional analysis for a particular gene has become more accessible through technical innovations in genome information science. Genomic markers for CpG islands, Histone methylation, and p300 ChIP-sequences gave us information that was useful for identifying the enhancer regions. Our results from reporter assays using deleted BACs or isolated enhancers agreed well with this genetic marker information.

Our present data indicate that D5E2 is a useful tool for identifying *Msi1-*expressing cells. Thus, transcriptional analysis of D5E2 has the potential to accelerate the elucidation of Msi1-mediated NS/PC and cancer stem cell maintenance. Interestingly, Msi1 has been reported as a marker for germinal cells in the intestine; our *Msi1*-reporter mouse may be useful for studying intestinal and other Msi1-expressing somatic stem cells [27]. Furthermore, Msi1 is expressed in the bulge of the hair follicles in which stem cells reside [30]. Interestingly, Nestin positive cells also exist in the bulge and the *Nestin second intron*-driven GFP positive/keratin 15 negative cells isolated from the bulge area can differentiate into neurons and Schwann cells *in vivo *[65-68]. Thus, common transcription factors could be involved in the expression of Msi1 and Nestin in the bulge area cells and NS/PCs. This possibility should be demonstrated in the future. Further analysis of *Msi1-*reporter mouse and the identification of *Msi1 *enhancers in other somatic stem cells may shed light on the regulation of stem cell maintenance.

## Conclusions

The D5E2 region on the sixth intron of the *Msi1 *gene is one of the most effective *Msi1 *enhancers. This enhancer region is especially active in NS/PCs and astrocytes, in which there is marked endogenous Msi1 expression.

## Methods

### Generation of the *Msi1-ffLuc *BAC clone

The mouse genomic BAC clone RP24-132L16 was obtained from the Children's Hospital of Oakland Research Institute (CHORI), and its derivatives were used for *Msi1 *transcription reporter assays. To generate the engineered BAC clone, *ffLuc *was combined with 300-bp regions homologous to the Msi1 gene at both the 5' and 3' flanking ends of *ffLuc*. This DNA combination was constructed on the selection cassette vector pL451, containing a neomycin-resistance gene with *FRT *sites. The homologous recombination selection cassette was linearized by SalI and PmeI digestion, gel-purified (Qiagen), and transformed into *E. Coli *DH10B with RP24-132L16 BAC DNA by standard electroporation methods (Gene Pulser Xcell, Bio-Rad). Recombination was performed with the Red ET system (Gene Bridges), and the cells were then cultured on LB medium plates containing chloramphenicol and kanamycin. Neo-resistant clones were analyzed by junctional sequence to identify correctly-targeted BACs. Through this procedure, *ffLuc *was inserted at the *Msi1 *transcriptional start site on the BAC DNA. The BAC DNA was purified using a Large-Construct Kit (Qiagen).

### Generation of the *Msi1-ffLuc *transgenic mouse

A *Flp *expression vector (706-Flp, Gene Bridges) was transformed into the *Msi1-ffLuc *BAC containing DH10B to remove the *pGKp-neo-GHp(A) *site (see general Gene Bridges method). Replacement was confirmed by sequencing and by kanamycin selection. DNA was linearized at a site within the vector with Pl-SceI, purified by gel chromatography, and injected into the pronucleus of fertilized mouse eggs. One expression line was established on a C57BL/6 background.

### Construction of D1-D5 BAC clones and minimized enhancer reporter genes

The D1-D5 deletion cassettes were constructed on pL452 using 300 bp of the homologous sites flanking the deletion on each side. Deleted clones were identified by kanamycin selection and by sequencing analysis of both sides of the deletion site from the cassette inserted into the BAC. Recombination was performed with a Red ET system.

The primer sets used to generate the deletion cassettes were as follows:

5' homology site

D1-D4: (5'-BstXIGACAGGCACTGTGAGAACCAAC-3' and 5'-NotICTTTACTGTCCGTCTGTCCGTC-3')

D5: (5'-BstXIGAAGGAGATCGTGGACTATGTGG-3' and 5'-NotIGCAATCGGATCCGAAGTTCCTATAC-3')

3' homology site

D1: (5'-EcoRIGGAGAGATGGCTCAGTGGTTAAGAG-3' and 5'-SalICCAGGAGCACTTGTCCAGAG-3')

D2: (5'-EcoRIGAGGTCAAGGATGCTAACTGTGC-3' and 5'-SalICTGAGCCTTGCTGTCTGGCTC-3')

D3: (5'-EcoRIGTAGCTGCCTGAAGTCCTGCATC-3'and 5'-SalIGAGACAGGGATCTCACTATGTAGC-3')

D4: (5'-EcoRIGACTCTGGTACACATGGGAGACC-3' and 5'-SalICACCACCGTCAGTGACCAC-3')

D5: (5'-EcoRI GACAGGCACTGTGAGAACCAAC-3' and 5'-SalICTTTACTGTCCGTCTGTCCGTC-3')Minimized enhancer reporter genes were constructed on PGV-P2; SV40 promoter and Luciferase alignment were replaced with an *Msi1 *promoter (P1) and *ffLuc*.

The primer sets used to generate D2E, D5E1, and D5E2 were as follows:

D2E: (5'-CTGTGGGTTATCTTGGGGAAATCTTC-3' and 5'-CCAGACAGCAAGGCTCAGG-3')

D5E1: (5'-GACCAGATCTTAGGAGACCCTG-3' and 5'-CAACCCCCTTATCAATCTTGGACG-3')

D5E2: (5'-GATCTGGGTCCAAGACGCAG-3' and 5'-CTCCTGAGGCTGGCTGAG-3')

### Introduction of recombinant BAC into ESCs

All BAC DNAs were linearized at a site within the vector using Pl-SceI. DNA (20 μg) was electroporated into 2 × 10^6 ^undifferentiated ESCs (EB3 tg14 line) [69]. Electroporation was performed in a Bio-Rad Gene Pulser set at 0.25 kV and 300 μF using a 0.4 cm gap cuvette. Electroporated cells were transferred to a 100-mm gelatinized dish containing 10 ml of GMEM medium (Sigma G6148) supplemented with 10% FBS, glutamine (2 mM), nonessential amino acids (0.1 mM), sodium pyruvate (1 mM), 2-mercaptoethanol (2-ME) (0.1 mM), sodium bicarbonate (3 mM), HEPES (5 mM), and mLIF. Selection was done with 300 μg/ml neomycin. After eight days of culture, colonies were picked up, transferred to a 24-well gelatinized plate, and allowed to expand in a complete medium.

### Neural induction of ESCs

For embryoid body (EB) formation, ESCs were detached and dissociated into single cells with 0.25% trypsin-EDTA, and were then transferred onto a non-coated bacteriological dish (Kord-Valmarkk) containing 10 ml of αMEM (Gibco 11900-024) supplemented with 10% FBS, sodium bicarbonate (3 mM), and 0.1 mM 2-ME (EB medium) at a density of 5 × 10^4 ^cells/ml. After 2 days in floating culture, 10^-8 ^M all-transretinoic acid (RA: Sigma R 2625) was added to the culture medium and EBs were cultured for 4 days.

### Neurosphere formation

EBs, including neural differentiated cells (NS/PCs), were collected on day 6 and were allowed to settle to the bottom of the tube for a few minutes. The collected EBs were washed once with PBS and incubated with 0.25% trypsin-EDTA for 5 min. The enzymatic reaction was quenched by adding an equal volume of EB medium, and the cells were dissociated with a transfer pipette by triturating 30 times. The cells were then washed twice with serum-free αMEM and resuspended in Media hormone mix (MHM) medium, which contains DMEM/F-12 (1:1) (Gibco 12100-046, 21700-075), glucose (0.6%), glutamine (2 mM), sodium bicarbonate (3 mM), HEPES (5 mM), insulin (25 Ag/ml), transferrin (100 Ag/ml), progesterone (20 nM), sodium selenate (30 ng), and putrescine (60 nM) (all from Sigma except for DMEM/F-12). The dissociated EBs were cultured in MHM supplemented with 20 ng/ml FGF and 20 ng/ml EGF for 6 days.

### Neuronal and glial differentiation of neural stem cells

The dissociated secondary neurospheres were plated on poly-l-ornithine/fibronectin-coated 10-mm cover glasses at a cell density of 1.5 × 10^5 ^cells/0.75 cm^2 ^on a 48-well culture plate and were allowed to differentiate for 4 days.

### Luciferase assays

For transient plasmid transfection, mouse day-14.5 embryo telencephalons were dissected, and the dissociated cells were cultured in MHM (+20 ng/ml FGF-2, +20 ng/ml EGF) for 5 days. After some passages, 1 × 10^5 ^cells were cultured for 2 days on 24-well culture plates, after which they were transfected with plasmids using GeneJuice (Novagen). After 2 days, the cells were washed with PBS, cell lysis buffer (Toyo Ink) was added, and the cells were incubated for 20 min at room temperature. Luciferase substrate solution (25 μl) (Promega) was added, and the luminescence was measured with a Berthold Centro LB960 Luminometer. The assays were internally calibrated with a standardized Renilla Luciferase solution to ensure uniformity between experiments. For stable transgenic ESCs, CellTiter-Blue (Promega) was used to count living cells for an internal control.

### FACS sorting and qRT-PCR

EBs were dissociated with trypsin-EDTA, resuspended in αMEM containing 10 mg/ml propidium iodide (PI), and filtered (30 μm). Cell sorting and analyses were performed using a FACS MoFlo flow cytometer/cell sorter equipped with CELLQuest software (Becton-Dickinson). Total RNA was isolated by the RNeasy Mini Kit (Qiagen) from cell fractions sorted according to GFP fluorescent intensity. Synthesis of cDNA was performed with Superscript II RNase H reverse transcriptase (Invitrogen) at 42°C for 50 min according to the manufacturer's instructions. Quantitative PCRs were performed with MX3000 (Stratagene). To analyze the relative expression of different mRNAs, the amount of cDNA was normalized to the level of ubiquitously expressed *α-actin *mRNA. The primer sets used were as follows:

*GFP: *(5'- TGAACCGCATCGAGCTGAAGGG-3' and 5'- TCCAGCAGGACCATGTGATCGC-3'),

*Msi1: *(5'-GGGATGGACGCCTTCATGCTG-3' and 5'-TGGCTTGGAACCCTGGGTAAC-3'),

*Nestin: *(5'-CTGAGAACTCTCGCTTGCAGACA-3' and 5'- GGAAATGCAGCTTCAGCTTGG-3').

### Immunohistochemistry

Frozen sections (12 μm) of brains fixed with 4% paraformaldehyde were prepared using a cryostat (CM3000, Leica). The sections were incubated with primary antibodies in TNB blocking buffer (PerkinElmer) at 4°C overnight, and then with fluorescent dye-conjugated secondary antibodies at room temperature for 1 hr. The images were observed by fluorescence microscopy (Axioplan2 Imaging, Carl Zeiss) and confocal laser scanning microscopy (LSM700, Carl Zeiss). The following antibodies were used: anti-Musashi1 (rat IgG) [9] (1:500), anti-Nestin (mouse IgG, BD) (1:200), anti-GFAP (rat IgG, Invitrogen) (1:200), anti-Group B1 SOX [SOX1/(2)/3] (1:5000), and anti-GFP (chick IgG, Aves) (1:500). Anti-Group B1 SOX [SOX1/(2)/3] antibody is reactive to SOX1 and SOX3 proteins, but it also weakly recognizes SOX2 protein [39].

## List of abbreviations

(Msi1): Musashi1; (FGF): fibroblast growth factor; (EGF): epidermal growth factor; (RA): retinoic-acid; (TSS): transcriptional start site; (NS/PCs): neural stem/progenitor cells; (ESCs): embryonic stem cells; (UTR): untranslated region; (CNS): central nervous system;

## Competing interests

The authors declare that they have no competing interests.

## Authors' contributions

SK and TI performed the experiments. SK and TI designed the experiments and analyzed data. CH and AM provided the *ffLuc *cDNA. KY and SI produced the *Msi1*-BAC transgenic animal. YM supported FACS experiment. SF participated in the preparation of this study. SK, TI, YN, and HO wrote the manuscript. HO provided financial support for the experiments. All authors have read and approved the manuscript.

## Supplementary Material

Additional file 1 **Figure S1. GFP-expressing cells in the central nervous system of adult *Msi1-ffLuc *transgenic mice**. Anti-GFP and anti-Msi1 immunoreactivities coincided well in the subgranular zone of the hippocampus (A) and the subependimal zone of the lateral wall of the lateral ventricle (B), where neurogenesis occurs. GFAP-positive neural stem cells in the subependimal zone were also GFP-positive (C). GFAP-positive astrocytes in the corpus callosum also showed GFP fluorescence (D). Scale bar: 20 μm. Click here for file

Additional file 2 **Figure S2. *Msi1-*reporter GFP levels correlate with endogenous Msi1 levels**. Day-6 EBs(+RA) were dissociated, fixed, and immunostained with anti-Msi1 and anti-GFP antibodies. The immunofluorescence intensity was then analyzed by flow cytometry. Of these cells, 80% were both GFP- and Msi1- positive (left panel). Right panel shows the negative control stained with secondary antibodies alone. The vertical axis shows FITC intensity; the horizontal axis shows PE intensity. Click here for file

Additional file 3 **Figure S3. H3K4 methylation status of the *Msi1 *enhancer regions in ESCs and ESC-derived NS/PCs**. ChIP-sequencing data was gathered from the UCSC genome browser (Broad H3 ChIP-sequence track). H3K4me1, known as an enhancer code for chromatin modification, intensely marked the D5E1 and D5E2 enhancer sites in NS/PCs. D3E was also H3K4me1-positive. D2E was strongly marked in ESCs and was also marked in NS/PCs. These three sites are highly conserved (see lower panel). Click here for file

Additional file 4 **Figure S4. D5E2 is an active enhancer in secondary neurosphere and tertialry neurosphere**. D5E2-P1 was transcriptionally active in secondary neurospheres and tertiary neurospheres. P1 alone was not active in either secondary neurospheres or tertiary neurospheres. Click here for file

Additional file 5 **Figure S5. D5E2 functions bi-directionally as an enhancer**. A reverse-oriented D5E2-P1 strand linked with the *ffLuc *3' end enhanced the transcriptional activity in ESCs and E14.5 NS/PCs [D5E2-P1 = 1 in each cell, D5E2 reverse-P1 = 0.4 1(ESCs), 0.56 (NS/PCs), P1 = 0.2 1(ESCs), 0.12 (NS/PCs)]. The data represent the mean ±SEM of three independent experiments. The data were subjected to non-repeated-measures ANOVA tests, and p values were calculated by Bonferroni multiple comparison tests. *p < 0.05: P1 to D5E2-P1, D5E2 reverse-P1. Click here for file

Additional file 6 **Figure S6. Candidate transcription-factor binding sites in D5E2**. Sequence comparisons of the *Msi1 *enhancer D5E2 sites between human, dog, mouse and rat species; three potential SOX and four potential AP-2 highly conserved binding sites were identified (searched results are from the JASPAR CORE database). Click here for file
