# Supplementary figures and images for: Identification of a novel intronic enhancer responsible for the transcriptional regulation of musashi1 in neural stem/progenitor cells
Source: Mol Brain. 2011 Apr 13;4:14. doi: 10.1186/1756-6606-4-14 (PMC3108301; doi:10.1186/1756-6606-4-14)

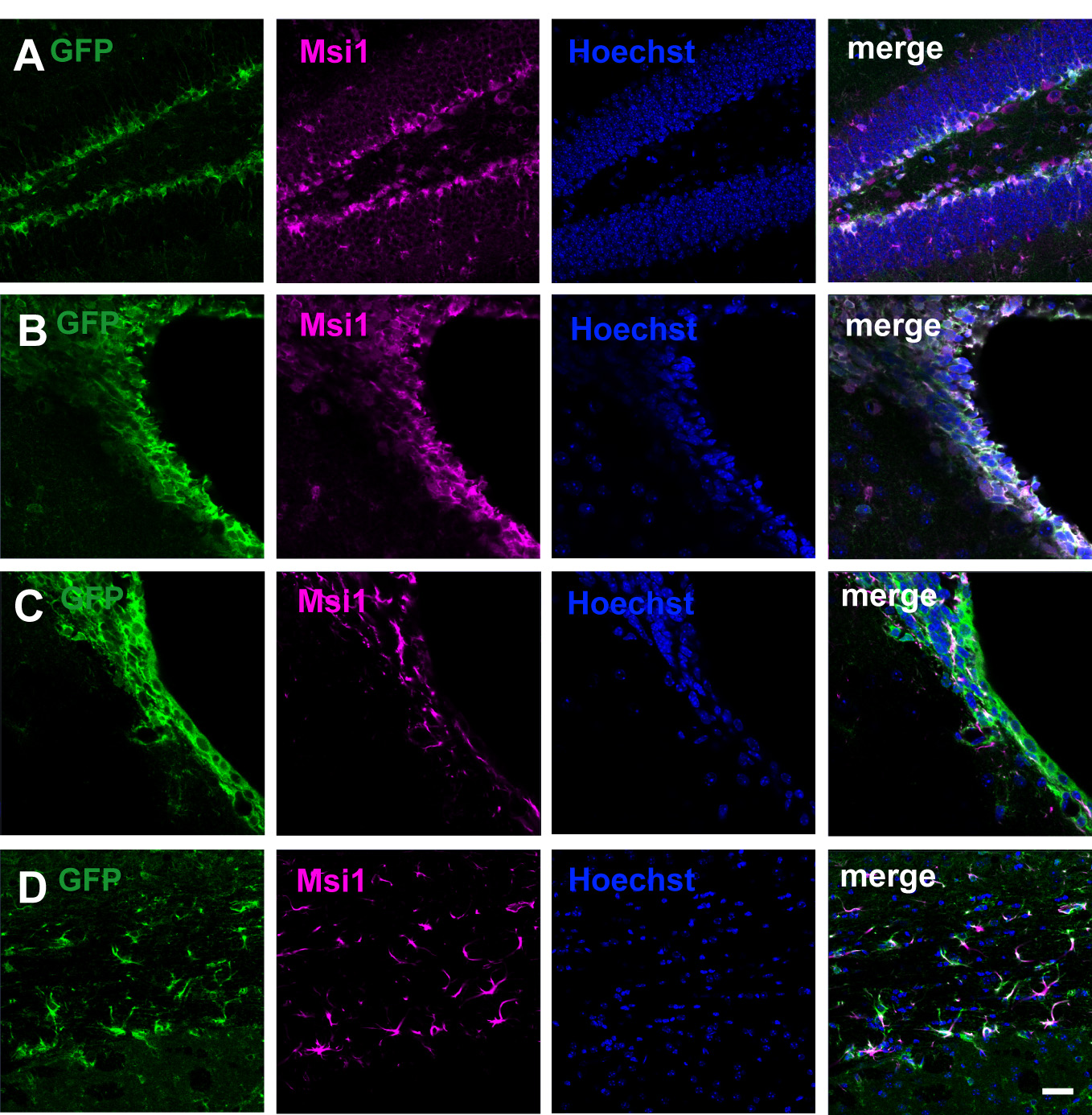

Supplement: Additional file 1 — Figure S1. GFP-expressing cells in the central nervous system of adult Msi1-ffLuc transgenic mice. Anti-GFP and anti-Msi1 immunoreactivities coincided well in the subgranular zone of the hippocampus (A) and the subependimal zone of the lateral wall of the lateral ventricle (B), where neurogenesis occurs. GFAP-positive neural stem cells in the subependimal zone were also GFP-positive (C). GFAP-positive astrocytes in the corpus callosum also showed GFP fluorescence (D). Scale bar: 20 μm. [file 1756-6606-4-14-S1.JPEG]

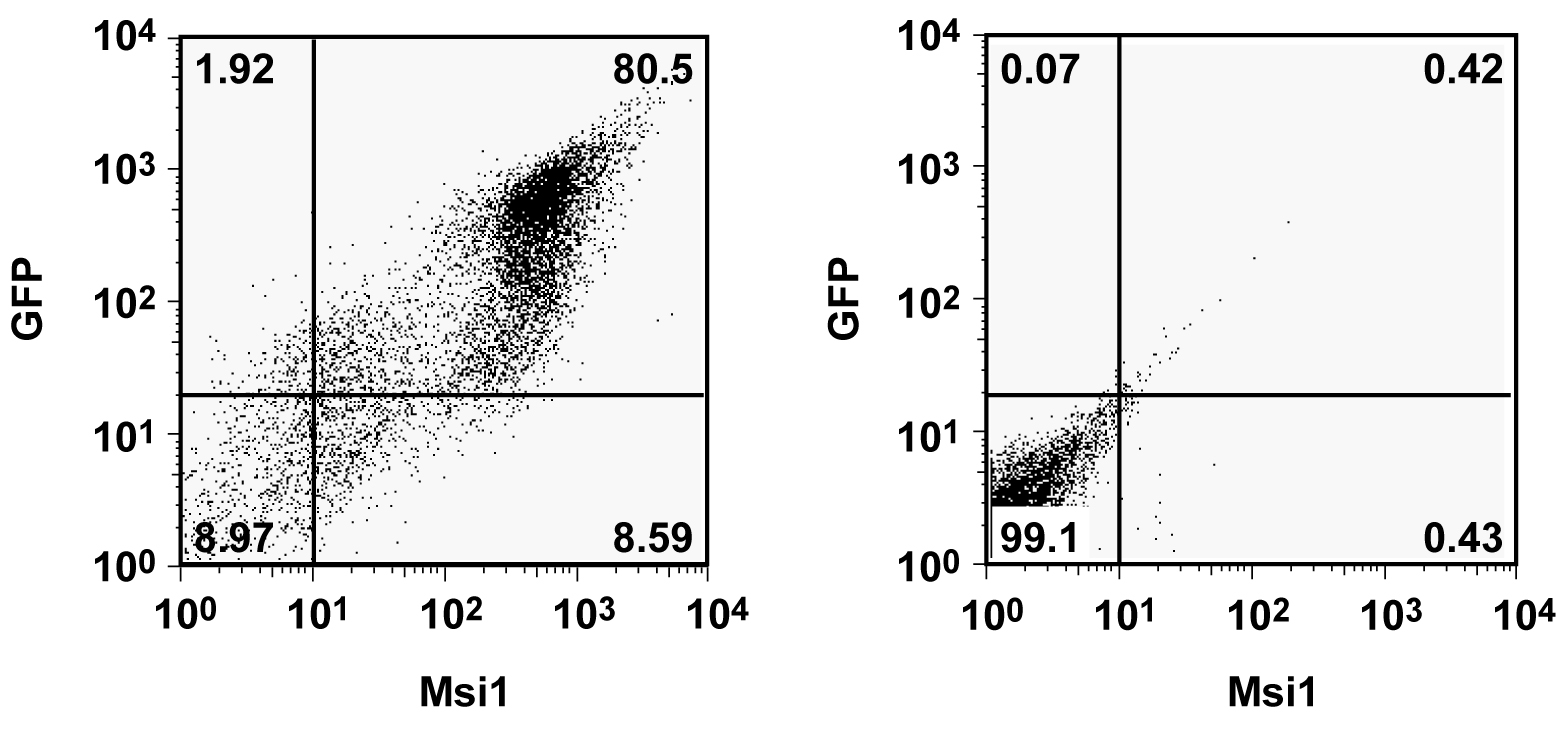

Supplement: Additional file 2 — Figure S2. Msi1-reporter GFP levels correlate with endogenous Msi1 levels. Day-6 EBs(+RA) were dissociated, fixed, and immunostained with anti-Msi1 and anti-GFP antibodies. The immunofluorescence intensity was then analyzed by flow cytometry. Of these cells, 80% were both GFP- and Msi1- positive (left panel). Right panel shows the negative control stained with secondary antibodies alone. The vertical axis shows FITC intensity; the horizontal axis shows PE intensity. [file 1756-6606-4-14-S2.JPEG]

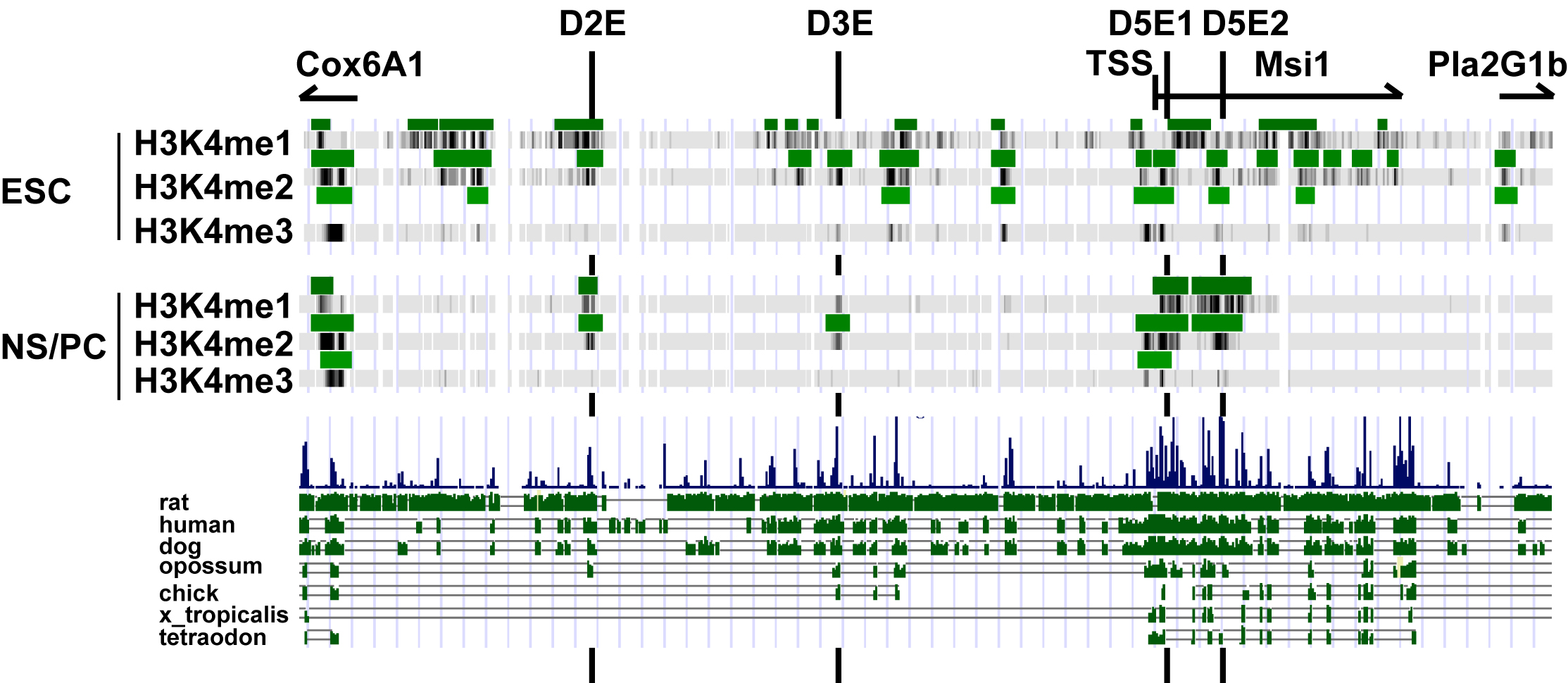

Supplement: Additional file 3 — Figure S3. H3K4 methylation status of the Msi1 enhancer regions in ESCs and ESC-derived NS/PCs. ChIP-sequencing data was gathered from the UCSC genome browser (Broad H3 ChIP-sequence track). H3K4me1, known as an enhancer code for chromatin modification, intensely marked the D5E1 and D5E2 enhancer sites in NS/PCs. D3E was also H3K4me1-positive. D2E was strongly marked in ESCs and was also marked in NS/PCs. These three sites are highly conserved (see lower panel). [file 1756-6606-4-14-S3.JPEG]

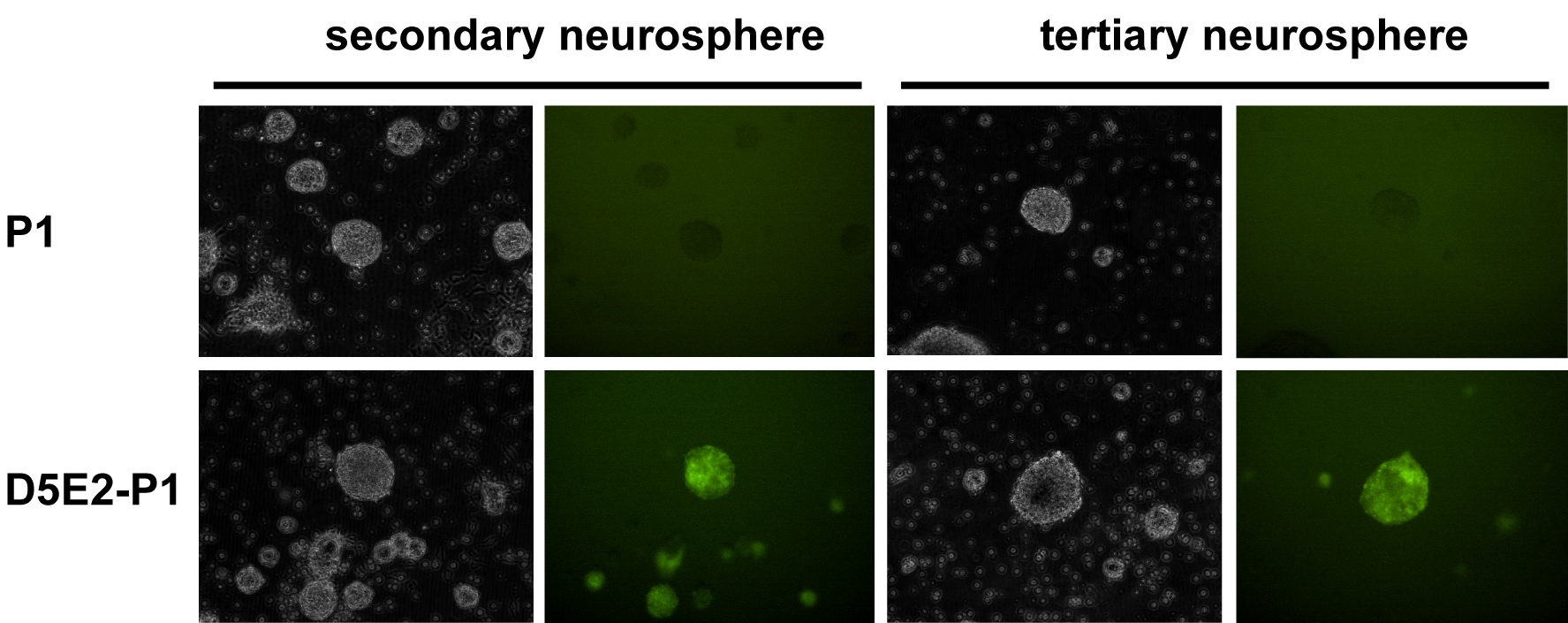

Supplement: Additional file 4 — Figure S4. D5E2 is an active enhancer in secondary neurosphere and tertialry neurosphere. D5E2-P1 was transcriptionally active in secondary neurospheres and tertiary neurospheres. P1 alone was not active in either secondary neurospheres or tertiary neurospheres. [file 1756-6606-4-14-S4.JPEG]

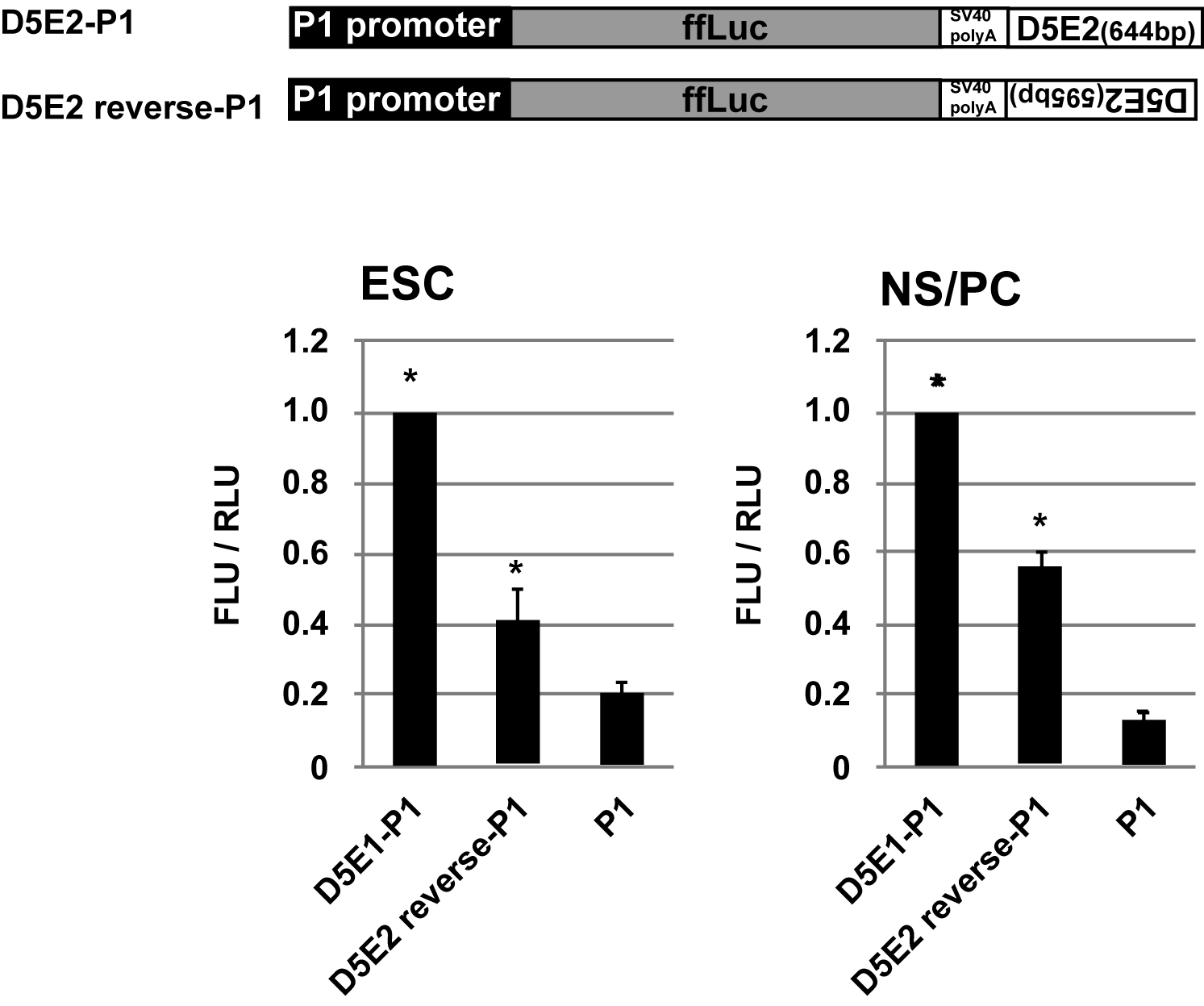

Supplement: Additional file 5 — Figure S5. D5E2 functions bi-directionally as an enhancer. A reverse-oriented D5E2-P1 strand linked with the ffLuc 3' end enhanced the transcriptional activity in ESCs and E14.5 NS/PCs [D5E2-P1 = 1 in each cell, D5E2 reverse-P1 = 0.4 1(ESCs), 0.56 (NS/PCs), P1 = 0.2 1(ESCs), 0.12 (NS/PCs)]. The data represent the mean ±SEM of three independent experiments. The data were subjected to non-repeated-measures ANOVA tests, and p values were calculated by Bonferroni multiple comparison tests. *p < 0.05: P1 to D5E2-P1, D5E2 reverse-P1. [file 1756-6606-4-14-S5.JPEG]

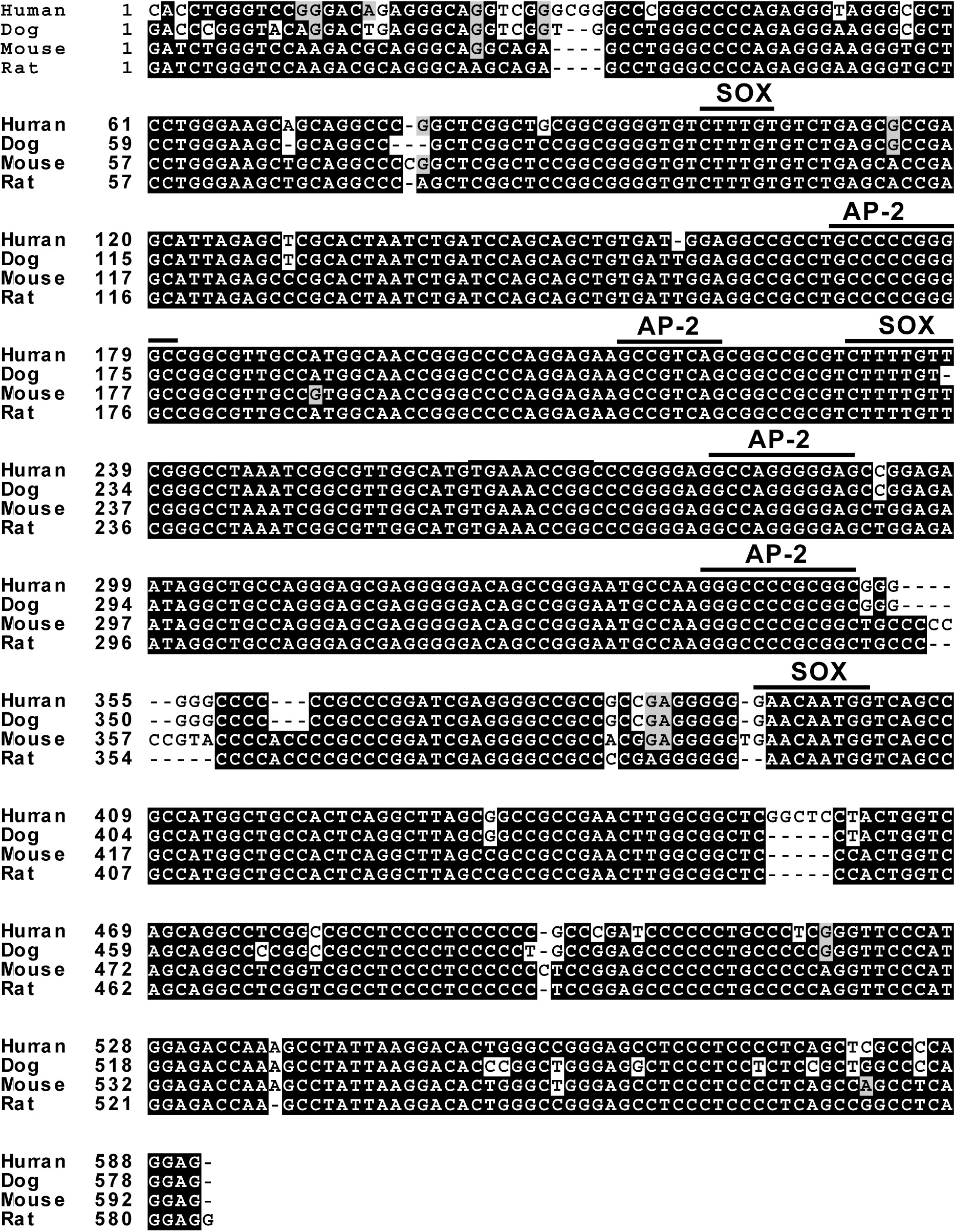

Supplement: Additional file 6 — Figure S6. Candidate transcription-factor binding sites in D5E2. Sequence comparisons of the Msi1 enhancer D5E2 sites between human, dog, mouse and rat species; three potential SOX and four potential AP-2 highly conserved binding sites were identified (searched results are from the JASPAR CORE database). [file 1756-6606-4-14-S6.JPEG]
